# Tumor hypoxia as a driving force in genetic instability

**DOI:** 10.1186/2041-9414-4-5

**Published:** 2013-10-24

**Authors:** Kaisa R Luoto, Ramya Kumareswaran, Robert G Bristow

**Affiliations:** 1 Ontario Cancer Institute, Radiation Medicine Program, Princess Margaret Cancer Centre (University Health Network), Toronto, ON, Canada; 2 Departments of Medical Biophysics and Radiation Oncology, University of Toronto, Radiation Medicine Program, Princess Margaret Cancer Centre (University Health Network), 610 University Avenue, Toronto, ON M5G2M9, Canada

**Keywords:** Hypoxia, Genetic instability, DNA damage, DNA double-strand breaks, DNA repair

## Abstract

Sub-regions of hypoxia exist within all tumors and the presence of intratumoral hypoxia has an adverse impact on patient prognosis. Tumor hypoxia can increase metastatic capacity and lead to resistance to chemotherapy and radiotherapy. Hypoxia also leads to altered transcription and translation of a number of DNA damage response and repair genes. This can lead to inhibition of recombination-mediated repair of DNA double-strand breaks. Hypoxia can also increase the rate of mutation. Therefore, tumor cell adaptation to the hypoxic microenvironment can drive genetic instability and malignant progression. In this review, we focus on hypoxia-mediated genetic instability in the context of aberrant DNA damage signaling and DNA repair. Additionally, we discuss potential therapeutic approaches to specifically target repair-deficient hypoxic tumor cells.

## Introduction

The tumor microenvironment is characterized by sub-regions of nutrient deprivation, low extracellular pH, high interstitial fluid pressure, and hypoxia. Hypoxic areas arise when oxygen consumption exceeds that of supply [1]. In normal tissues, the oxygen supply matches the metabolic requirements of the cells. However, in locally advanced solid tumors, the oxygen consumption increases significantly, resulting in inadequate oxygen supply to some regions of the tumor. In addition, the blood vessels within a tumor microenvironment are usually chaotic, dilated and irregularly organized [1]. In normal tissues, the oxygen tension (pO_2_) ranges from 10 to 80 mmHg (or 1.25% to 10% O_2_). However, tumors often contain regions where the oxygen concentration can significantly decrease to less than 5 mmHg (or < 0.6% O_2_) [2,3]. Clinical studies using pO_2_ electrodes, hypoxia imaging (positron emission tomography (PET)), and immunohistochemistry (IHC) have demonstrated that hypoxia is a characteristic of all solid tumors [4]. Hypoxic regions within tumors can be measured by IHC assessment of intrinsic and extrinsic hypoxic cell biomarkers. Intrinsic biomarkers of hypoxic response include hypoxia inducible factor 1 (HIF1α), vascular endothelial growth factor (VEGF), carbonic anhydrase IX (CAIX), osteopontin and glucose transporters 1 and 3 (GLUT1, GLUT3) and the extrinsic biomarkers include drugs that specifically accumulate or become bio-reduced to form adducts within hypoxic cells such as pimonidazole (PIMO), EF5 and CCI-103 F [5]. Increased levels of hypoxia correlates with genetic instability, tumor progression, local and systemic resistance; all leading to poor clinical outcome following treatment [6-12].

Tumor cells that lie beyond the diffusion distance for oxygen (> 70 μm away from blood vessels) can quickly outstrip blood supply and are exposed to chronically low oxygen tensions [13]. These diffusion-limited conditions for duration of days are referred to as “prolonged” or “chronic hypoxia” [14]. The cells in these regions are believed to remain hypoxic until they die (due to lack of oxygen or nutrients) or are reoxygenated [15]. Hypoxia can also be transient or “cycling” due to acute perfusion changes in the tumor vasculature. The blood vessels formed during unregulated angiogenesis contain severe structural and functional abnormalities and can temporarily close and re-open, leading to cycles of acute hypoxia/anoxia (from minutes to hours) followed by reoxygenation (hence, cycling hypoxia) [14]. Both acute and chronic hypoxia co-exist within a tumor resulting in significant gradients of oxygen consumption leading to intratumor heterogeneity [16].

In an experimental setting, cellular hypoxia can be induced by placing cultured tumor cells in complete media in environmentally-controlled chambers in which oxygen levels in the gas phase are maintained at 0.01-3% [17]. These hypoxic conditions may not be lethal nor growth inhibitory to selected tumor cell lines when cultured in the presence of excess glucose and nutrients. However, when cells are placed in the complete absence of oxygen (anoxia), most cells will stop proliferating due to the activation of anoxia-mediated intra-S phase arrest mediated by the ataxia telangiectasia mutated (ATM) and ataxia telangiectasia and RAD3-related (ATR) kinases [18-21]. If prolonged, this arrest of DNA replication becomes irreversible leading to cell death mechanisms [22]. Hence, a permanent anoxic microenvironment (e.g. close to 0% O_2_) eventually leads to cell death whereas tumor cells that exist in hypoxic microenvironments (e.g. 0.2 to 1% O_2_) could adapt and continue to proliferate with altered biology [12,14]. Tumor cells that adapt to low oxygen conditions gain an overall advantage for growth and leads to treatment resistance following chemotherapy or radiotherapy [14]. Therefore, the study of proliferating hypoxic cells is important as it represents a clinically-challenging, sub-population of resistant cells with the potential of clonal expansion and metastatic spread.

Clinical observations, supported by pre-clinical data, have demonstrated that hypoxia is associated with an increased capacity for metastasis [23]. Metastasis is a multi-step process that involves disruption of cell adhesion to the neighboring cells and to the basement membrane, migration through the extracellular matrix, penetration of vessel walls and circulation exit, and finally initiation of angiogenesis to allow tumor growth in the target tissue [24]. Hypoxia can lead to altered expression of many proteins involved in this process by regulating the expression of E-cadherin (cell-cell contact), urokinase-type plasminogen activator receptor (uPAR; degradation of extracellular matrix proteins), hepatocycte growth factor (HGF; cellular motility) and vascular endothelial growth factor (VEGF; angiogenesis and vascular permeability) [14,24,25]. Hypoxia also limits the effectiveness of many anti-cancer therapies. The efficiency of ionizing radiation to create lethal DNA breaks is strongly associated with oxygen tension and creation of free radicals. Oxygen can react with the damaged DNA bases created by free radicals to yield a more stable adduct and this reaction chemically “fixes” the damage [2]. Indeed, oxygenated cells can be two to three times more sensitive to radiation than hypoxic or anoxic cells [12,26]. However, ionizing radiation under anoxic conditions has been shown to increase the levels of DNA-protein crosslinks [27,28]. Moreover, poor drug distribution and decreased proliferation can decrease the efficacy of many chemotherapy drugs [12,14]. Thus, the cells in hypoxic regions can adapt to become resistant to radiotherapy and chemotherapy and ongoing selection of increasing aggressiveness [29]. Therefore, two main clinical entities are associated with hypoxic tumors: increased local tumor cell resistance and development of systemic metastasis. Despite these data, hypoxia-targeted therapy is still not a standard of current cancer treatments [30]. Therefore, the study of hypoxic cells is important in order to gain a further understanding of the consequences of the hypoxic microenvironment for the development of genetic instability as a precursor to tumor progression and therapy-associated resistance.

## Hypoxia-mediated genetic instability

**Figure 1:**
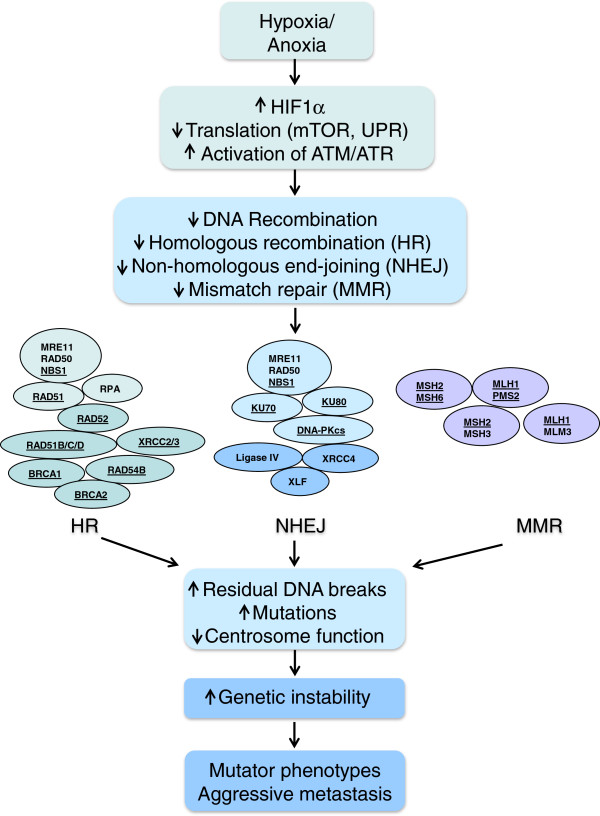
**Mechanism(s) of hypoxia**-**driven genetic instability.** Hypoxia/anoxia signalling and subsequent adaptive biology is mediated by HIF1α transcription factors and altered protein through the unfolded protein response (UPR). These transcriptional and translational responses inhibit DNA repair by homologous recombination, non-homologous end-joining, and mismatch repair. The proteins downregulated by hypoxia are underlined. As a result, increased unrepaired double-strand breaks, replication errors and decreased centrosome function can accelerate genetic instability and lead to an aggressive, mutator phenotype.

Tumor cells can acquire multiple adaptations in the selective pressure of the tumor microenvironment. Hypoxia inducible factor 1α (HIF1α) is a transcription factor, which is kept at low levels in the presence of oxygen by von Hippel-Lindau protein (VHL)-mediated degradation [31]. In hypoxic conditions, HIF1α is quickly stabilized and regulates a number of genes including those involved in vascularization, glycolysis and pH homeostasis [31]. HIF1α is crucial for hypoxic adaptation, and overexpression of HIF1α is associated with a poor disease outcome [32]. Loss of HIF1α control can promote the malignant phenotype and genomic instability via interplay with oncoproteins such as c-MYC [33-37]. Oncogene amplification, DNA replication stress, and deregulated DNA damage checkpoint signaling in hypoxic tumor cells, together with the ability to escape cell death, can allow cells to proliferate in the presence of damaged DNA and acquire further mutations [38,39]. The vicious cycle is accelerated by increased frequency of mutations and by the ability of hypoxic cells to downregulate DNA repair; therefore further driving genomic instability (see Figure 1) [14,40]. Moreover, when hypoxic cells become reoxygenated, they may acquire further DNA damage as a result of a sudden burst of free radicals [41,42]. We now discuss further hypoxia-mediated genomic instability in the context of the DNA damage signaling and inhibited DNA repair.

## Hypoxia and the DNA Damage Response (DDR): checkpoints and DNA replication

Human cells maintain genetic integrity by detecting DNA damage and activating cell cycle checkpoints and DNA repair pathways [43]. The G1/S, intra-S, and the G2/M checkpoints, are mediated by ATM/ATR and checkpoint kinases 2 and 1 (CHK2)/(CHK1), respectively [18-21,43]. These kinases transmit signals to the effector molecules p53, p21 (G1/S) and CDC25 (G1/S, intra-S and G2/M) to prevent cell cycle progression or to initiate programmed cell death [44,45]. Cycles of hypoxia followed by reoxygenation in tumors cyclically activates many DNA damage response (DDR) proteins. Furthermore, ATM, DNA-PKcs, H2AX, p53, CHK1, CHK2, 53BP1 and NBS1 are phosphorylated under conditions of severe hypoxia (<0.02%) in the absence of exogenous DNA damage [18,41,46-51]. Anoxia therefore leads to cell cycle arrests at G1 and intra-S in the absence of DNA damage, and in turn, reoxygenation causes CHK2-mediated G2 arrest [12,19,21,22,38,52]. When an arrested hypoxic cell becomes reoxygenated, it may either resume proliferation or undergo an irreversible loss of DNA replication ability and undergo cell death [38,53-55]. The length of the hypoxic stress may determine the ultimate fate of a cancer cell [38]. Cell cycle changes however depend on the level of hypoxia. For example, oxygen levels such as 0.2% do not activate ATM or ATR and cell cycle checkpoint signaling [56]. Propagation of such a tumor cell with potentially altered DNA damage signaling and reoxygenation-induced DNA damage, can contribute to genetic instability and malignant progression [38].

HIF1α can also bind directly to minichromosome maintenance (MCM) proteins that are responsible for unwinding the DNA during replication [57]. Direct interaction between HIF1α and MCM7 results in increased prolyl hydroxylation-dependent HIF1α degradation, and an interaction with MCM3 results in HIF1α transactivation domain function inhibition [58]. HIF1α can block replication origin firing and DNA replication by binding to Cdc6, which is involved in recruiting MCM helicases to replication origins. HIF1α-Cdc6 interaction leads to enhanced MCM helicase loading and decreased recruitment of Cdc7 to replication origins, resulting inhibition of replication origin firing and overall DNA replication [57].

## Hypoxia causes microsatellite and chromosomal instability

Studies have also documented an increased rate of spontaneous DNA mutations in cells exposed to hypoxia using reporter assays. This further supports the view of tumor microenvironment as a driving force of genomic instability (see Table 1) [59-62]. The concept of genetic instability covers a wide variety of genetic alterations from point mutations to chromosomal number. These changes are divided into two types: microsatellite instability (MSI) and chromosomal instability [63]. MSI is typically found in colorectal cancers and is caused by defective DNA mismatch repair (MMR) [64]. As hypoxia downregulates MMR, a model of tumor microenvironment-driven MSI has been proposed. This suggestion is supported by studies both in vitro and in vivo of colorectal cancer models [65-67]. High level of HIF1α associates with MSI in human colorectal carcinoma [68,69]. Further investigation in clinical settings will show whether the mechanistic laboratory findings of HIF-MMR-MSI can be generalized to other cancers in addition to colon carcinomas.

**Table 1:** Evidence linking hypoxia to tumor cell genetic instability

**Author**	**% Oxygen**	**Cell system**	**Assays**	**Key findings**
Rice et al. [70]	0%	AA8 (CHO)	Flow cytometry, gene copy analyses	- Anoxia induces S-phase overreplication and increases the frequency of dihydrofolate reductase gene amplification
Young et al. [71]	0% (<10 ppm)	KHT-C2-LP1 (M-fibrosarcoma),	Metastasis assay, flow cytometry	- Anoxia induces DNA overreplication and increases metastatic potential
		B16F10-A1 (M-melanoma)		
Reynolds et al. [61]	0% (<10 ppm)	LN12 (M-fibroblasts)	Chromosome based λ shuttle vector, PCR, DNA sequence analysis	- Anoxia induces 3–4 fold increase in *supF* tRNA suppressor gene mutation (transversions and deletions) frequency
Rofstad et al. [72]	0% (<10 ppm and <100 ppm)	BEX-c (H-melanoma),	Flow cytometry, Giemsa	- Anoxia followed by reoxygenation induces diplochromosomes and tetraploidization
		SAX-c (H-melanoma)		
Coquelle et al. [73]	0.02%	GMA32 (Chinese hamster fibroblasts)	Fluorescence in situ hybridization (FISH)	- Severe hypoxia induces fragile sites and generates homogeneously stained regions (HSRs)
Yuan et al. [74]	0% (<10 ppm)	3340 (M-fibroblast)	Host cell reactivation (HCR) assay, UV mutagenesis assay	- Anoxia induces 2-fold increase in *supFG1* mutation frequency
Coquelle et al. [75]	0.02%	GMA32 (Chinese hamster fibroblasts), 112 (Chinese hamster fibroblasts)	Fluorescence in situ hybridization (FISH)	- Severe hypoxia activates fragile sites and generates double minutes and dicentric chromosomes
Mihaylova et al. [76]	0% <10 ppm	3340 (M-fibroblasts), HeLa (H-cervix adenocarcinoma), EMT6 (M-breast carcinoma)	β-galactosidase and *supFG1* mutation assays	- Anoxia induces 2-fold increase in *supFG1*, *cII* and *lacZ* mutation frequency
Banath et al. [77]	i.p. pimonidazole	V79-VE (Chinese hamster fibroblasts),	Flow cytometry, γ-H2AX foci, HPRT mutation assay, alkaline comet assay	- Hypoxia (cells distant to the blood vessels) followed by reoxygenation does not alter mutation frequency at HPRT locus, DNA strand break rejoining or resolution of γ-H2AX foci following ionizing radiation (IR)
		HCT116 (H-colon carcinoma),		
		SCCVII (M-squamous cell carcinoma)		
Koshiji et al. [78]	1%	HCT116 (H-colon carcinoma),	β-galactosidase mutation assay, microsatellite analysis	- Hypoxia increases the frequency of microsatellite mutations
		HEC59 (H-endometrial carcinoma)		
Papp-Szabo et al. [59]	0%	ME (R-mammary epithelial cells),	*cII* mutagenicity assay	- Anoxia increases the mutation frequency by 2-fold at *cII* locus without affecting colonogenic survival
		MFib (R-mammary fibroblasts)		
Fischer et al. [79]	0%	TX3868 (H-glioblastoma)	Fluorescence in situ hybridization (FISH)	- Anoxia induces double minutes, fragile sites and anaphase-bridges and initiates gene amplification on chromosome 12q
Rodriguez-Jimenez et al. [80]	1%	C17.2 (M-multipotent neural precursor cells), M-primary neurospheres from CD31, BMMSC (H-mesenchymal stem cells), DPSC (H-mesenchymal stem cells)	Host cell reactivation (HCR) assay, microsatellite instability analysis	- Hypoxia increases mutation frequency of the β-galactosidase reporter gene and causes microsatellite instability
Keysar et al. [60]	<0.1%	A_L_(N) (CHO)	Complement cytotoxic assay, flow cytometry mutation assay	- Anoxia results in a significant induction of mutations especially large deletions in *CD59* gene
Lee et al. [81]	3%	Primary lymphocytes from healthy donors	Sister chromatid exchange (SCE) assay, microsatellite instability assay	- Hypoxia increases SCE but does not alter microsatellite instability
Pires et al. [38]	<0.02%	RKO (H-colon carcinoma), HCT116 (H-colon carcinoma), U2OS (H-osteosarcoma), IBR3 (H-fibroblast)	DNA fiber analysis, immunofluorescence	- Anoxia blocks DNA replication at the initiation and elongation stages and compromises DNA replication restart - Acute anoxia following reoxygenation (cycling hypoxia) does not affect DNA replication restart
Kumareswaran et al.* [82]	0.2%	GM05757 (H-fibroblasts)	Giemsa, Multicolor fluorescence in situ hybridization (M-FISH)	- Hypoxia increases the frequency of fragmented DNA, ring chromosomes, telomeric fusions, chromosomal translocations and marker chromosomes following exogenous DNA damage

CHO – Chinese hamster ovary cells; M – mouse; H – human; R – rat.

*only study investigating DNA repair under continual hypoxic conditions.

DNA double-strand break (DSB) repair is crucial for chromosomal integrity. Unrepaired DSBs can lead to formation of deletions, insertions, translocations and amplifications [83,84]. For example, cells deficient for BRCA1/2 develop spontaneous gross chromosomal aberrations [85-89]. Hypoxia is known to both inhibit DSB repair and to promote chromosomal instability in multiple ways [71,73,90]. Fragile sites are specific chromosomal regions prone to chromosomal breakage and rearrangements during replication stress and are induced under hypoxia [73,91]. This could be, in part, explained by hypoxia-mediated downregulation of DSB repair genes, as RNAi inhibition of DSB repair results in fragile site activation [92]. Additionally, ATM and ATR kinases maintain fragile site stability, and DSB biomarkers γ-H2AX and DNA-PKcs^Thr2609^ foci localize at fragile sites [92,93]. An unrepaired DSB can also lead to DNA amplification, which has been observed in hypoxic cells [70,71,75,79,94]. Additionally, the frequency of sister chromatid exchange (SCE), which is in part controlled by homologous recombination (HR) repair, may be increased in hypoxic primary human lymphocytes [81,95]. Human fibroblasts subjected to continual hypoxic conditions following exogenous DNA damage maintained increased chromosomal aberrations such as chromosome breaks, chromatid breaks, ring chromosomes, telomeric fusions, reciprocal translocations and double minutes [82]. Finally, hypoxia may also induce global deacetylation and methylation of histones, phosphorylation of H2AX and altered condensation states within the chromatin [90].

In order to prevent mitotic errors leading to genetic instability, the cell must properly align chromosomes during mitosis. The mitotic spindle is generated by the activity of centrosomes, which are composed of centrioles and pericentriolar material [96]. Defects in centrosomes and spindle formation lead to aneuploidy during the process of carcinogenesis and tumor progression [97,98]. Recently, a study has shown that hypoxia can modify centrosome function by altering the activity of prolyl-4-hydroxylases (PHDs) towards the protein Cep192 (a critical component of the centrosome) [99]. This allows for mediating signaling between oxygen tension and cell cycle control. Further studies are required to investigate whether these and other genes that are involved in mitosis and centrosome organization are altered in cancer cells within hypoxic sub-regions of solid tumors.

Altogether, these studies support the concept that hypoxia can modify fragile sites, the repair of DNA damage, chromatin biology, and possibly mitosis in promoting genetic instability during tumor progression.

## Hypoxia-mediated inhibition of DNA repair

The understanding of hypoxia in the context of signaling and DNA repair is increasing based on data using isogenic models that vary in specific DNA repair pathways. Below, we discuss the mechanisms of DNA repair downregulation in hypoxic cells in a pathway-specific manner (Figure 1).

### DNA double-strand break repair

Ionizing radiation (IR) or radiomimetic drugs create DSBs, which are mainly repaired by HR or non-homologous end-joining (NHEJ) pathways in a cell cycle-dependent manner [100]. The proteins RAD51, BRCA1/2 and the MRN complex (MRE11, RAD50, NBS1) together regulate HR during S and G2 phases of the cell cycle. Proteins such as KU70/80, DNA-PKcs and DNA-ligase IV function in NHEJ across all phases of the cell cycle [100].

The majority of HR proteins are repressed by chronic hypoxia [101]. This can occur through decreased transcription, translation, miRNA modulation and epigenetic silencing. The first mechanistic model suggests that HIF1α competes with and opposes MYC activity in hypoxic cells, inhibiting *Brca1* and *Nbs1* transcription [35,102-104]. Another model proposes that HR gene expression, including *Rad51* and *Brca1*, is repressed by the E2F-4/p130 complex independent of HIF [105-107]. The HIF-independent mechanism is supported by observations of downregulated RAD51 in isogenic HIF1α^−/−^ mouse embryo fibroblasts (MEFs) under hypoxia, albeit by reduced efficiency [108]. Studies from our laboratory support a third model involving selective inhibition of protein synthesis. Hypoxia alters protein synthesis by pathways that modulate gene expression in both transcript-specific and a global manner; via unfolded protein response (UPR) and mammalian target of rapamycin (mTOR) signaling [109]. Our findings indicate that in chronically hypoxic proliferating cells, RAD51 and BRCA2 are downregulated due to selective inhibition of mRNA translation [56]. Yet another layer to hypoxia-regulated HR expression involves altered chromatin modification and *Brca1* promoter silencing in severe hypoxia [110]. Finally, miRNA may play a role in HR suppression and can affect *Rad52* gene expression [111].

The impact of hypoxia and DNA repair on malignant progression is demonstrated in studies indicating that repressed HR is linked with cancer initiating cell formation [112]. Breast tumor-initiating cells overexpress polycomb protein EZH2, which is further induced by HIF1α under hypoxia [112,113]. EZH2 inhibits *Rad51* transcription in hypoxic CD44^+^CD24^-/low^ cells, which is associated with increased genomic abnormality [112]. This EZH2-RAD51 signaling (via RAF1 amplification) promotes mammosphere formation and malignant progression [112].

**Figure 2:**
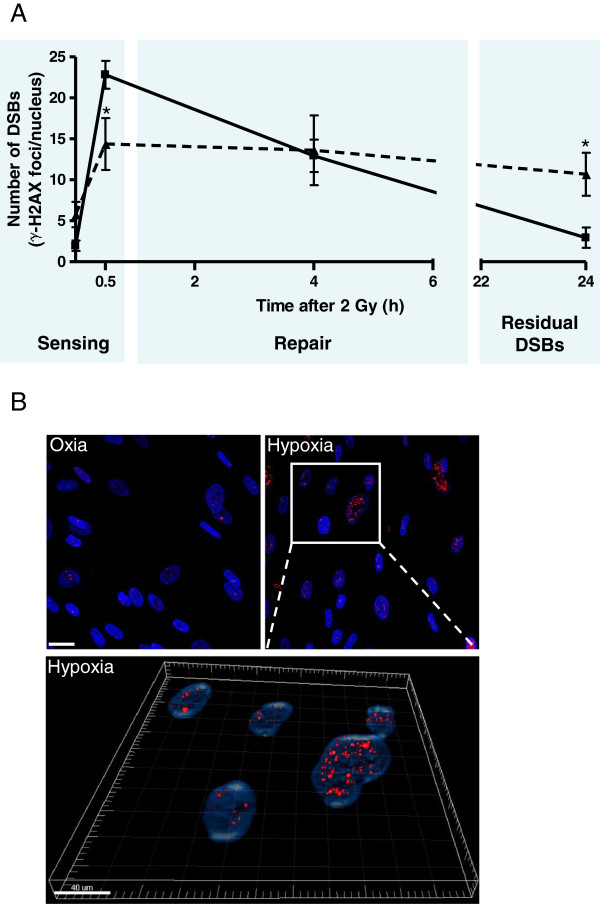
**Decreased repair of DNA double strand breaks (DNA**-**DSBs) under continual hypoxia. A**, Despite a decrease in the initial number of induced and sensed DSBs measured by γ-H2AX foci at 30 minutes following 2 Gy, hypoxic (0.2% O_2_) G0/G1 synchronized human fibroblasts have an increased number of residual γ-H2AX foci at 24 hours. The asterisk represents a significant difference (*P < 0.05) between oxic control (solid) and hypoxic treatment (dashed). Plot is adapted from data published in Kumareswaran et al. [82]. **B**, Two dimensional (top panels) and three dimensional (bottom panel) confocal images of G0/G1 fibroblasts with increased number of residual γ-H2AX foci under continual hypoxia at 24 hours following 2 Gy of irradiation. Scale bar = 10 μm.

**Figure 3:**
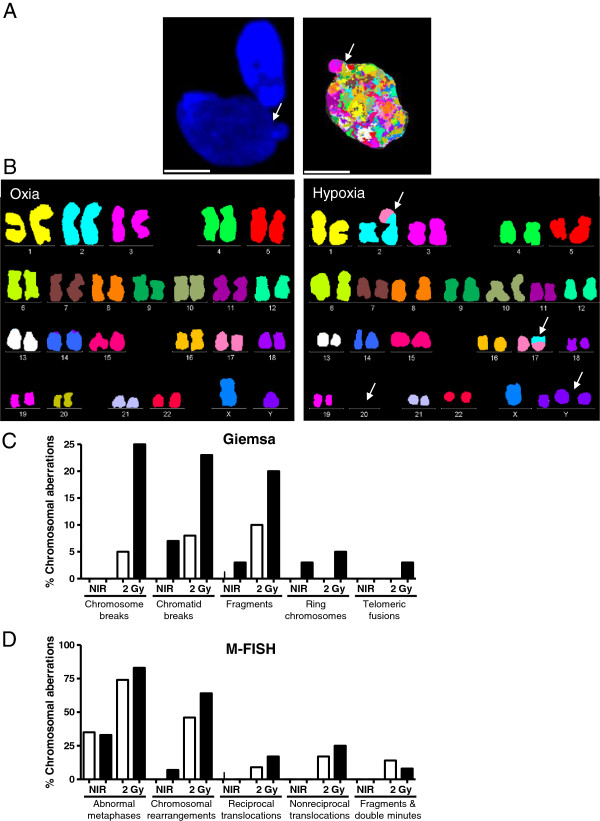
**Hypoxia induces chromosomal aberrations following exogenous damage. A**, Chromatin bridges or anaphase bridges in fibroblasts maintained under continual hypoxic (0.2% O_2_) conditions following 2 Gy of irradiation. These bridges can break into fragments and give rise to micronuclei [121]. The type, the number, and the fate of chromosome bridges under hypoxia is not known and requires further investigation. Representative DAPI stained and M-FISH images of fibroblasts are shown. Scale bar = 10 μm. **B**, M-FISH karyotype of fibroblasts maintained under oxic (21% O_2_) conditions following 2 Gy of irradiation or hypoxic (0.2% O_2_) conditions following 2 Gy of irradiation. Shown are reciprocal translocation between chromosomes 2 and 17, loss of chromosome 20 and two extra copies of chromosome Y in hypoxic cells following 2 Gy of irradiation. **C**, Percentages of chromosomal aberrations in oxic and hypoxic fibroblasts as measured by Giemsa staining analysis. NIR = non-irradiated; white columns = oxia (21% O_2_); black columns = hypoxia (0.2% O_2_). **D**, Percentages of chromosomal aberrations in oxic and hypoxic fibroblasts as measured by M-FISH analysis. NIR = non-irradiated; white columns = oxia (21% O_2_); black columns = hypoxia (0.2% O_2_). Plots are based on quantitative assessment of data published in Kumareswaran et al. [82].

The function of NHEJ in hypoxia-driven genetic instability and radiation response is more controversial. Inhibited expression of *DNA-PKcs*, *Ku70, Ku80* and *DNA-ligase IV* has been observed under hypoxia [101,114]. NHEJ factors are downregulated in hypoxic wild-type MEFs and in normoxic HIF1α^−/−^ MEFs [115]. In cervical tumors, KU70/KU80 expression correlates with oxygen pressure and is inhibited with increasing distance to blood vessels [116]. We observed an increase in residual DSBs in G0/G1 synchronized human fibrobalsts under hypoxic conditions following exogenous DNA damage (Figures 2 and 3) [82]. On the other hand, induction of *Ku70* may occur under hypoxia in some cell lines [114]. KU70 could indeed contribute to hypoxic tumor cell resistance to radiation, as expression of a dominant negative form of KU70 sensitizes hypoxic glioma and colorectal cells to radiation [117]. Other reports have proposed redundancy or increased NHEJ under hypoxia [118-120]. An outstanding question in the field is whether the MRN complex, ATM and DNA-PKcs kinases differentially sense DSBs under oxia vs hypoxia (Figure 1). Varying model systems and tumor microenvironment conditions might explain the differing observations, and further investigation will clarify the role of hypoxia in NHEJ control.

### Mismatch repair

MMR repairs DNA base substitutions and misalignments, which occur during DNA replication [122]. Mammalian MMR uses proteins such as MutSα (MSH2 + MSH6), MutSβ (MSH2 + MSH3), and MutLα (MLH1 + PMS2) [122].

The involvement of MMR in the hypoxic response is fairly well characterized. The hypoxia-driven genetic instability in colorectal cancers is consistent with inhibited *Mlh1* transcription in low oxygen [76]. Mechanistically, MMR inhibition under hypoxia involves at least MYC and DEC transcription factors. Interplay of HIF1α and MYC has been suggested to regulate MMR expression; MYC-dependent regulation of MSH2 and MSH6 in oxic cells may be replaced by HIF1α under hypoxia [35,78,104]. In addition, knockdown of HIF1α reverses hypoxia-driven inhibition of MMR expression [78,123]. Repression of MMR gene expression by decreased MYC and increased MAX, MAD and MNT association on *Mlh1* and *Msh2* promoters have been observed in hypoxic cells [107]. MYC, MAD and MNT (as part of the “max network” containing basic helix-loop-helix zipper (bHLHZ) motifs) form heterodimers with MAX resulting in sequence-specific DNA binding [124]. These DNA-bound heterodimers can then alter chromatin structure to modulate transcription [124]. Additionally, hypoxia-induced transcription repressors DEC1 and DEC2 contribute to *Mlh1* inhibition [125]. Hypoxic MMR regulation is also influenced by the state of chromatin acetylation [66,76,80,125].

### Nucleotide excision repair and Fanconi anemia pathway

Chemicals covalently bound to DNA forming bulky adducts, as well as chemical-caused DNA crosslinks and UV-induced DNA lesions, are repaired by nucleotide excision repair (NER). NER in mammals uses two pathways: global genome repair (GGR) and transcription-coupled repair (TCR) [126]. GGR involves multiple sequential steps including sensing of the lesion (XPC-HR23B-Centrin 2 complex), opening of a denaturation bubble (TFIIH, XPA-RPA complex), incision of damaged strand (XPG, XPF-ERCC1 complex), displacement of lesion-containing oligonucleotides and gap filling (DNA Pol δ and ϵ) and ligation (ligase III, ligase I) [126]. On the other hand, TCR requires CSA, CSB and XAB2 to sense the lesion and proceeds to GGR for the next sequential steps [126]. Both decreased and increased ability of cells to repair UV-damaged DNA in conditions of hypoxia and low pH have been reported [74,120]. Indication for NER in the hypoxic response comes from findings of XPC and XPD as direct HIF1α targets, and inhibition of HIF1α perturbs the removal of UVB-induced 6–4 photoproducts (6-4PPs) and cyclobutane pyrimidine dimers (CPDs) [127]. Also, HIF1α associates with the gene promoter of CSB/ERCC6, which functions in recruiting NER repair proteins to the damaged DNA, and is induced by hypoxia. CSB mutant cells fail to activate HIF-dependent hypoxic response [128]. Finally, RAD23B protein is repressed under hypoxia and by miRNA-373 [111]. Further investigation is needed to establish the role of hypoxia in NER.

Fanconi anemia (FA) is a hereditary disorder with predisposition to cancer [129]. The FA pathway includes 14 FANC genes, which function in ubiquitination-phosphorylation pathways and participate in repairing DNA interstrand crosslinks created by agents such as (mitomycin C) MMC or cisplatin [129]. Little is known regarding the role of FANC in the hypoxic response. However, FANCC and FANCD2 cells exhibit increased IR sensitivity under hypoxia compared to wild-type cells [118,130]. UBE2T is an E2 conjugating enzyme that operates in the FA pathway to mono-ubiquitinate FANCD2 and FANCI. UBE2T expression is inhibited under hypoxia by a mechanism involving decreased promoter activity, independent of HIF1α, HIF1β or HIF2α. Consistent with the FA phenotype, both anoxic and UBE2T knockdown cells are hypersensitive to MMC-induced DNA crosslinks [131].

## Therapeutic targeting of hypoxic tumor cells

**Figure 4:**
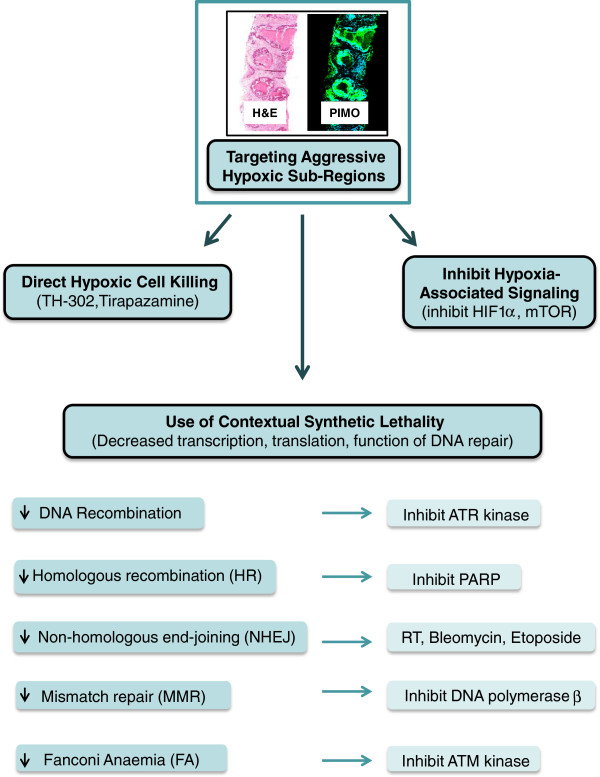
**Targeting of hypoxic cells in cancer treatment.** Hypoxic cells can be quantitated in situ by staining for antibodies that measure uptake of nitroimidazole compounds (which are reduced in hypoxic environments and bind to SH-containing molecules such as glutathione and proteins); one such compound is pimonidazole (PIMO). These studies, in addition to direct measurements of pO_2_, have linked the proportion of hypoxic cells to aggressive tumor cell variants that are resistant to radiotherapy, chemotherapy and have an increased propensity for metastases. Direct targeting with agents that create DNA damage solely under hypoxic conditions (e.g. TH-302) or inhibit selective pathways activated in hypoxic cells (e.g. HIF1α and mTOR signaling) may improve the overall cell kill within a tumor volume when used alone or with radiotherapy or chemotherapy. Hypoxia may also lead to differential transcription or translation of DNA repair or replication genes which can reduce the function of the repair pathway. These repair-deficient hypoxic cells can be killed by agents that target remaining back-up pathways leading to cell death. Given the repair defect is secondary to the effects of hypoxia as opposed to a primary somatic or germline defect, this type of cell kill is denoted, “contextual synthetic lethality” given it is contextual on the local tumor microenvironment and varies depending on the metabolic state of the cancer cell.

The success of anti-cancer therapies is currently challenged by increased local and systemic resistance of tumor cells residing in the hypoxic microenvironment. However, the hypoxic phenotype can also provide an opportunity to specifically target cells in the tumor microenvironment and improve the therapeutic index (e.g. kill more cancer cells than normal cells) (see Figure 4). The development of therapeutic agents that are selectively activated upon exposure to low oxygen is of great interest [32]. For example, tirapazamine and apaziquone, both bioreductive prodrugs that induce DNA damage, have been tested in Phase III clinical trials [32]. A newer compound, TH-302, is a 2-nitroimidazole triggered hypoxia-activated prodrug of the cytotoxin bromo-isophosphoramide mustard (Br-IPM), which causes DNA damage under hypoxic/anoxic conditions [132]. The antitumor activity of TH-302 has been shown to be dose-dependent and decreased the hypoxic fraction in xenografts of varying histology. TH-302 also induces DNA damage (as measured by γ-H2AX) in hypoxic regions in vivo and can further kill cells through a time-dependent “bystander effect”. This compound is currently in Phase II-III clinical trials in combination with chemotherapy.

Translational control is an important contributor to the hypoxic adaptation and gene expression alongside with HIF-dependent pathways [109]. Therefore, targeting mTOR and UPR could provide another opportunity to enhance selective tumor cell kill [32,133,134]. Clinically relevant agents that influence mTOR or UPR signaling include for example imatinib, nelfinavir and sunitinib, which can improve tumor oxygenation and inhibit angiogenesis [109,135].

Synthetic lethality is a phenomenon that arises when mutations in two or more genes result in cell death, while a cell with a mutation in either gene alone is viable [136]. Over the recent years, this has started to attract attention as a way to attack the Achilles’ heel of a cancer cell. For example, inhibition of poly(ADP-ribose) polymerase (PARP), which normally functions in single-strand break (SSB) and base-excision repair (BER), is synthetically lethal with BRCA-deficient tumors [137]. In addition to targeting cancerous mutations, synthetic lethality based on tumor microenvironment has emerged, where the extrinsic differences of tumor cells are used to widen the therapeutic index [136]. In this “contextual” synthetic lethality, the hypoxic phenotype with defective DNA repair can be exploited, together with inhibiting a backup DNA repair pathway, to specifically kill hypoxic cells. Therapies would therefore preferentially kill tumor cells with reduced DNA repair capacity, and spare normal tissue with physiological oxygenation state and functional DNA repair. Indeed, hypoxic HR-defective cells are sensitive to PARP inhibition [108,138]. PARP inhibition induces DNA damage in proliferating cells and kills hypoxic cells specifically in S phase [108]. Synthetic lethality in the HR pathway has also been documented between RAD52 and BRCA2, as well as between splicing factor proline and glutamate-rich (SFPQ)/PSF and RAD51D [139,140]. Additionally, PTEN null astrocytes were found to be sensitive to PARP inhibition due to lower expression of *Rad51B-D*[141]. However, recent data from our laboratory failed to observe a correlation between PTEN status and RAD51 function [142].

In MMR, inhibition of POLB in MSH2-deficient; and inhibition of POLG in MLH1-deficient cells, produces a synthetic lethal phenotype [143]. An siRNA screen identified inhibited PTEN-induced putative kinase 1 (PINK1) as lethal in cells deficient in MLH1, MSH2 and MSH6 [144]. Given that most HR factors and MMR are downregulated under hypoxia, determining whether these synthetic lethal interactions could be exploited to target hypoxic tumor cells, would be of great interest. Future investigations will show if these observations could have an impact on radiation- and clinical oncology.

## Conclusions

A number of molecular mechanisms have been proposed to explain hypoxic inhibition of HR and MMR-mediated DNA repair based on biochemical and cell biology endpoints. Molecular pathways may play differing roles depending on tissue type, microenvironment conditions and proliferation status; or alternatively, each might have a relative contribution for a global DNA repair-deficient phenotype. Dissecting these pathways could help designing anti-cancer treatments that inhibit DNA repair and sensitize tumor cells to radio- and chemotherapies. Also, a better understanding of therapies targeting the proliferating hypoxic cell subpopulations could increase selective killing of resistant tumor cells. Clinical trials using these approaches will require careful assessment of the tumor microenvironment using imaging or other techniques in order to incorporate hypoxia assessment as a part of a standard of care. This approach will serve well to be one step closer to individualized cancer medicine and improved patient outcome.

## Competing interests

The authors declare that they have no competing interests.

## Authors’ contributions

KRL, RK and RGB wrote the manuscript. All authors read and approved the final manuscript.

## References

[B1] Vaupel P Harrison L Tumor hypoxia: causative factors, compensatory mechanisms, and cellular response Oncologist 2004 4 Suppl 5 4 9 15591417 10.1634/theoncologist.9-90005-4

[B2] Hill RP Bristow RG Tannock IF, Hill RP, Bristow RG, Harrington L The Scientific Basis of Radiotherapy The Basic Science of Oncology 2005 New York: McGraw-Hill Ltd 289 321

[B3] Chan N Koch CJ Bristow RG Tumor hypoxia as a modifier of DNA strand break and cross-link repair Curr Mol Med 2009 4 401 410 10.2174/156652409788167050 19519397

[B4] Vaupel P Mayer A Hypoxia in cancer: significance and impact on clinical outcome Cancer Metastasis Rev 2007 4 225 239 10.1007/s10555-007-9055-1 17440684

[B5] Ljungkvist AS Bussink J Kaanders JH van der Kogel AJ Dynamics of tumor hypoxia measured with bioreductive hypoxic cell markers Radiat Res 2007 4 127 145 10.1667/RR0719.1 17390721

[B6] Hockel M Knoop C Schlenger K Vorndran B Baussmann E Mitze M Knapstein PG Vaupel P Intratumoral pO2 predicts survival in advanced cancer of the uterine cervix Radiother Oncol 1993 4 45 50 10.1016/0167-8140(93)90025-4 8438086

[B7] Hockel M Schlenger K Aral B Mitze M Schaffer U Vaupel P Association between tumor hypoxia and malignant progression in advanced cancer of the uterine cervix Cancer Res 1996 4 4509 4515 8813149

[B8] Fyles A Milosevic M Hedley D Pintilie M Levin W Manchul L Hill RP Tumor hypoxia has independent predictor impact only in patients with node-negative cervix cancer J Clin Oncol 2002 4 680 687 10.1200/JCO.20.3.680 11821448

[B9] Knocke TH Weitmann HD Feldmann HJ Selzer E Potter R Intratumoral pO2-measurements as predictive assay in the treatment of carcinoma of the uterine cervix Radiother Oncol 1999 4 99 104 10.1016/S0167-8140(99)00139-5 10665785

[B10] Lyng H Sundfor K Trope C Rofstad EK Disease control of uterine cervical cancer: relationships to tumor oxygen tension, vascular density, cell density, and frequency of mitosis and apoptosis measured before treatment and during radiotherapy Clin Cancer Res 2000 4 1104 1112 10741740

[B11] Nordsmark M Bentzen SM Rudat V Brizel D Lartigau E Stadler P Becker A Adam M Molls M Dunst J Prognostic value of tumor oxygenation in 397 head and neck tumors after primary radiation therapy. An international multi-center study Radiother Oncol 2005 4 18 24 10.1016/j.radonc.2005.06.038 16098619

[B12] Chan N Bristow RG “Contextual” synthetic lethality and/or loss of heterozygosity: tumor hypoxia and modification of DNA repair Clin Cancer Res 2010 4 4553 4560 10.1158/1078-0432.CCR-10-0527 20823145

[B13] Vaupel P The role of hypoxia-induced factors in tumor progression Oncologist 2004 4 Suppl 5 10 17 15591418 10.1634/theoncologist.9-90005-10

[B14] Bristow RG Hill RP Hypoxia and metabolism. Hypoxia, DNA repair and genetic instability Nat Rev Cancer 2008 4 180 192 10.1038/nrc2344 18273037

[B15] Rofstad EK Galappathi K Mathiesen B Ruud EB Fluctuating and diffusion-limited hypoxia in hypoxia-induced metastasis Clin Cancer Res 2007 4 1971 1978 10.1158/1078-0432.CCR-06-1967 17360973

[B16] Hoogsteen IJ Marres HA van der Kogel AJ Kaanders JH The hypoxic tumour microenvironment, patient selection and hypoxia-modifying treatments Clin Oncol (R Coll Radiol) 2007 4 385 396 10.1016/j.clon.2007.03.001 17433637

[B17] Papandreou I Powell A Lim AL Denko N Cellular reaction to hypoxia: sensing and responding to an adverse environment Mutat Res 2005 4 87 100 10.1016/j.mrfmmm.2004.06.054 15603754

[B18] Bencokova Z Kaufmann MR Pires IM Lecane PS Giaccia AJ Hammond EM ATM activation and signaling under hypoxic conditions Mol Cell Biol 2009 4 526 537 10.1128/MCB.01301-08 18981219 PMC2612523

[B19] Freiberg RA Hammond EM Dorie MJ Welford SM Giaccia AJ DNA damage during reoxygenation elicits a Chk2-dependent checkpoint response Mol Cell Biol 2006 4 1598 1609 10.1128/MCB.26.5.1598-1609.2006 16478982 PMC1430245

[B20] Gibson SL Bindra RS Glazer PM CHK2-dependent phosphorylation of BRCA1 in hypoxia Radiat Res 2006 4 646 651 10.1667/RR0660.1 17007555

[B21] Olcina M Lecane PS Hammond EM Targeting hypoxic cells through the DNA damage response Clin Cancer Res 2010 4 5624 5629 10.1158/1078-0432.CCR-10-0286 20876254 PMC3000384

[B22] Pires IM Bencokova Z McGurk C Hammond EM Exposure to acute hypoxia induces a transient DNA damage response which includes Chk1 and TLK1 Cell Cycle 2010 4 2502 2507 20581459 10.4161/cc.9.13.12059 PMC3040847

[B23] Subarsky P Hill RP The hypoxic tumour microenvironment and metastatic progression Clin Exp Metastasis 2003 4 237 250 10.1023/A:1022939318102 12741682

[B24] Sullivan R Graham CH Hypoxia-driven selection of the metastatic phenotype Cancer Metastasis Rev 2007 4 319 331 10.1007/s10555-007-9062-2 17458507

[B25] Chaudary N Hill RP Hypoxia and metastasis Clin Cancer Res 2007 4 1947 1949 10.1158/1078-0432.CCR-06-2971 17404073

[B26] Spiro IJ Rice GC Durand RE Stickler R Ling CC Cell killing, radiosensitization and cell cycle redistribution induced by chronic hypoxia Int J Radiat Oncol Biol Phys 1984 4 1275 1280 10.1016/0360-3016(84)90332-8 6469750

[B27] Murray D Meyn RE Vanankeren SC Variations in the spectrum of lesions produced in the DNA of cells from mouse tissues after exposure to gamma-rays in air-breathing or in artificially anoxic animals Int J Radiat Biol Relat Stud Phys Chem Med 1988 4 921 933 10.1080/09553008814551291 3259562

[B28] Zhang H Koch CJ Wallen CA Wheeler KT Radiation-induced DNA damage in tumors and normal tissues. III. Oxygen dependence of the formation of strand breaks and DNA-protein crosslinks Radiat Res 1995 4 163 168 10.2307/3579024 7724730

[B29] Chan N Milosevic M Bristow RG Tumor hypoxia, DNA repair and prostate cancer progression: new targets and new therapies Future Oncol 2007 4 329 341 10.2217/14796694.3.3.329 17547528

[B30] Overgaard J Hypoxic radiosensitization: adored and ignored J Clin Oncol 2007 4 4066 4074 10.1200/JCO.2007.12.7878 17827455

[B31] Semenza GL Hypoxia-inducible factors in physiology and medicine Cell 2012 4 399 408 10.1016/j.cell.2012.01.021 22304911 PMC3437543

[B32] Wilson WR Hay MP Targeting hypoxia in cancer therapy Nat Rev Cancer 2011 4 393 410 10.1038/nrc3064 21606941

[B33] Camps C Buffa FM Colella S Moore J Sotiriou C Sheldon H Harris AL Gleadle JM Ragoussis J hsa-miR-210 Is induced by hypoxia and is an independent prognostic factor in breast cancer Clin Cancer Res 2008 4 1340 1348 10.1158/1078-0432.CCR-07-1755 18316553

[B34] Fu L Wang G Shevchuk MM Nanus DM Gudas LJ Generation of a mouse model of Von Hippel-Lindau kidney disease leading to renal cancers by expression of a constitutively active mutant of HIF1alpha Cancer Res 2011 4 6848 6856 10.1158/0008-5472.CAN-11-1745 21908555 PMC3214086

[B35] Yoo YG Christensen J Huang LE HIF-1alpha confers aggressive malignant traits on human tumor cells independent of its canonical transcriptional function Cancer Res 2011 4 1244 1252 10.1158/0008-5472.CAN-10-2360 21303984 PMC3041864

[B36] Nakada C Tsukamoto Y Matsuura K Nguyen TL Hijiya N Uchida T Sato F Mimata H Seto M Moriyama M Overexpression of miR-210, a downstream target of HIF1alpha, causes centrosome amplification in renal carcinoma cells J Pathol 2011 4 280 288 10.1002/path.2860 21465485

[B37] Doe MR Ascano JM Kaur M Cole MD Myc Posttranscriptionally Induces HIF1 Protein and Target Gene Expression in Normal and Cancer Cells Cancer Res 2012 4 949 957 10.1158/0008-5472.CAN-11-2371 22186139 PMC3288382

[B38] Pires IM Bencokova Z Milani M Folkes LK Li JL Stratford MR Harris AL Hammond EM Effects of acute versus chronic hypoxia on DNA damage responses and genomic instability Cancer Res 2010 4 925 935 10.1158/0008-5472.CAN-09-2715 20103649 PMC2923514

[B39] Zafarana G Ishkanian AS Malloff CA Locke JA Sykes J Thoms J Lam WL Squire JA Yoshimoto M Ramnarine VR Copy number alterations of c-MYC and PTEN are prognostic factors for relapse after prostate cancer radiotherapy Cancer 2012 4 4053 4062 10.1002/cncr.26729 22281794

[B40] Huang LE Bindra RS Glazer PM Harris AL Hypoxia-induced genetic instability–a calculated mechanism underlying tumor progression J Mol Med 2007 4 139 148 10.1007/s00109-006-0133-6 17180667

[B41] Hammond EM Dorie MJ Giaccia AJ ATR/ATM targets are phosphorylated by ATR in response to hypoxia and ATM in response to reoxygenation J Biol Chem 2003 4 12207 12213 10.1074/jbc.M212360200 12519769

[B42] Hsieh CH Shyu WC Chiang CY Kuo JW Shen WC Liu RS NADPH oxidase subunit 4-mediated reactive oxygen species contribute to cycling hypoxia-promoted tumor progression in glioblastoma multiforme PLoS One 2011 4 e23945 10.1371/journal.pone.0023945 21935366 PMC3174133

[B43] O”Driscoll M Jeggo PA The role of double-strand break repair - insights from human genetics Nat Rev Genet 2006 4 45 54 16369571 10.1038/nrg1746

[B44] Shimada M Nakanishi M DNA damage checkpoints and cancer J Mol Histol 2006 4 253 260 10.1007/s10735-006-9039-4 16841236

[B45] Li L Zou L Sensing, signaling, and responding to DNA damage: organization of the checkpoint pathways in mammalian cells J Cell Biochem 2005 4 298 306 10.1002/jcb.20355 15578575

[B46] Hammond EM Denko NC Dorie MJ Abraham RT Giaccia AJ Hypoxia links ATR and p53 through replication arrest Mol Cell Biol 2002 4 1834 1843 10.1128/MCB.22.6.1834-1843.2002 11865061 PMC135616

[B47] Gibson SL Bindra RS Glazer PM Hypoxia-induced phosphorylation of Chk2 in an ataxia telangiectasia mutated-dependent manner Cancer Res 2005 4 10734 10741 10.1158/0008-5472.CAN-05-1160 16322218

[B48] Freiberg RA Krieg AJ Giaccia AJ Hammond EM Checking in on hypoxia/reoxygenation Cell Cycle 2006 4 1304 1307 10.4161/cc.5.12.2811 16760660

[B49] Bouquet F Ousset M Biard D Fallone F Dauvillier S Frit P Salles B Muller C A DNA-dependent stress response involving DNA-PK occurs in hypoxic cells and contributes to cellular adaptation to hypoxia J Cell Sci 2011 4 1943 1951 10.1242/jcs.078030 21576354

[B50] Harding SM Coackley C Bristow RG ATM-dependent phosphorylation of 53BP1 in response to genomic stress in oxic and hypoxic cells Radiother Oncol 2011 4 307 312 10.1016/j.radonc.2011.05.039 21680038

[B51] Kim BM Choi JY Kim YJ Woo HD Chung HW Reoxygenation following hypoxia activates DNA-damage checkpoint signaling pathways that suppress cell-cycle progression in cultured human lymphocytes FEBS Lett 2007 4 3005 3012 10.1016/j.febslet.2007.05.053 17544403

[B52] Gardner LB Li Q Park MS Flanagan WM Semenza GL Dang CV Hypoxia inhibits G1/S transition through regulation of p27 expression J Biol Chem 2001 4 7919 7926 10.1074/jbc.M010189200 11112789

[B53] Gardner LB Li F Yang X Dang CV Anoxic fibroblasts activate a replication checkpoint that is bypassed by E1a Mol Cell Biol 2003 4 9032 9045 10.1128/MCB.23.24.9032-9045.2003 14645516 PMC309642

[B54] Wang L Gao J Dai W Lu L Activation of Polo-like kinase 3 by hypoxic stresses J Biol Chem 2008 4 25928 25935 10.1074/jbc.M801326200 18650425 PMC2533803

[B55] Tan C Zhang LY Chen H Xiao L Liu XP Zhang JX Overexpression of the human ubiquitin E3 ligase CUL4A alleviates hypoxia-reoxygenation injury in pheochromocytoma (PC12) cells Biochem Biophys Res Commun 2011 4 403 408 10.1016/j.bbrc.2011.11.054 22120631

[B56] Chan N Koritzinsky M Zhao H Bindra R Glazer PM Powell S Belmaaza A Wouters B Bristow RG Chronic hypoxia decreases synthesis of homologous recombination proteins to offset chemoresistance and radioresistance Cancer Res 2008 4 605 614 10.1158/0008-5472.CAN-07-5472 18199558

[B57] Hubbi ME Kshitiz Gilkes DM Rey S Wong CC Luo W Kim DH Dang CV Levchenko A Semenza GL A nontranscriptional role for HIF-1alpha as a direct inhibitor of DNA replication Sci Signal 2013 4 ra10 10.1126/scisignal.2003417 23405012 PMC4124626

[B58] Hubbi ME Luo W Baek JH Semenza GL MCM proteins are negative regulators of hypoxia-inducible factor 1 Mol Cell 2011 4 700 712 10.1016/j.molcel.2011.03.029 21658608 PMC3131976

[B59] Papp-Szabo E Josephy PD Coomber BL Microenvironmental influences on mutagenesis in mammary epithelial cells Int J Cancer 2005 4 679 685 10.1002/ijc.21088 15849743

[B60] Keysar SB Trncic N Larue SM Fox MH Hypoxia/reoxygenation-induced mutations in mammalian cells detected by the flow cytometry mutation assay and characterized by mutant spectrum Radiat Res 2010 4 21 26 10.1667/RR1838.1 20041756 PMC2848454

[B61] Reynolds TY Rockwell S Glazer PM Genetic instability induced by the tumor microenvironment Cancer Res 1996 4 5754 5757 8971187

[B62] Li CY Little JB Hu K Zhang W Zhang L Dewhirst MW Huang Q Persistent genetic instability in cancer cells induced by non-DNA-damaging stress exposures Cancer Res 2001 4 428 432 11212225

[B63] Michor F Iwasa Y Vogelstein B Lengauer C Nowak MA Can chromosomal instability initiate tumorigenesis? Semin Cancer Biol 2005 4 43 49 10.1016/j.semcancer.2004.09.007 15613287

[B64] Geiersbach KB Samowitz WS Microsatellite instability and colorectal cancer Arch Pathol Lab Med 2011 4 1269 1277 10.5858/arpa.2011-0035-RA 21970482

[B65] Shahrzad S Quayle L Stone C Plumb C Shirasawa S Rak JW Coomber BL Ischemia-induced K-ras mutations in human colorectal cancer cells: role of microenvironmental regulation of MSH2 expression Cancer Res 2005 4 8134 8141 10.1158/0008-5472.CAN-05-0713 16166287

[B66] Edwards RA Witherspoon M Wang K Afrasiabi K Pham T Birnbaumer L Lipkin SM Epigenetic repression of DNA mismatch repair by inflammation and hypoxia in inflammatory bowel disease-associated colorectal cancer Cancer Res 2009 4 6423 6429 10.1158/0008-5472.CAN-09-1285 19638594 PMC2748849

[B67] Kondo A Safaei R Mishima M Niedner H Lin X Howell SB Hypoxia-induced enrichment and mutagenesis of cells that have lost DNA mismatch repair Cancer Res 2001 4 7603 7607 11606400

[B68] Furlan D Sahnane N Carnevali I Cerutti R Bertoni F Kwee I Uccella S Bertolini V Chiaravalli AM Capella C Up-regulation of the hypoxia-inducible factor-1 transcriptional pathway in colorectal carcinomas Hum Pathol 2008 4 1483 1494 10.1016/j.humpath.2008.02.013 18619649

[B69] Lehtonen HJ Makinen MJ Kiuru M Laiho P Herva R van Minderhout I Hogendoorn PC Cornelisse C Devilee P Launonen V Aaltonen LA Increased HIF1 alpha in SDH and FH deficient tumors does not cause microsatellite instability Int J Cancer 2007 4 1386 1389 10.1002/ijc.22819 17520677

[B70] Rice GC Hoy C Schimke RT Transient hypoxia enhances the frequency of dihydrofolate reductase gene amplification in Chinese hamster ovary cells Proc Natl Acad Sci U S A 1986 4 5978 5982 10.1073/pnas.83.16.5978 3461470 PMC386420

[B71] Young SD Marshall RS Hill RP Hypoxia induces DNA overreplication and enhances metastatic potential of murine tumor cells Proc Natl Acad Sci U S A 1988 4 9533 9537 10.1073/pnas.85.24.9533 3200838 PMC282788

[B72] Rofstad EK Johnsen NM Lyng H Hypoxia-induced tetraploidisation of a diploid human melanoma cell line in vitro Br J Cancer Suppl 1996 4 S136 S139 8763866 PMC2149977

[B73] Coquelle A Toledo F Stern S Bieth A Debatisse M A new role for hypoxia in tumor progression: induction of fragile site triggering genomic rearrangements and formation of complex DMs and HSRs Mol Cell 1998 4 259 265 10.1016/S1097-2765(00)80137-9 9734364

[B74] Yuan J Narayanan L Rockwell S Glazer PM Diminished DNA repair and elevated mutagenesis in mammalian cells exposed to hypoxia and low pH Cancer Res 2000 4 4372 4376 10969780

[B75] Coquelle A Rozier L Dutrillaux B Debatisse M Induction of multiple double-strand breaks within an hsr by meganucleaseI-SceI expression or fragile site activation leads to formation of double minutes and other chromosomal rearrangements Oncogene 2002 4 7671 7679 10.1038/sj.onc.1205880 12400009

[B76] Mihaylova VT Bindra RS Yuan J Campisi D Narayanan L Jensen R Giordano F Johnson RS Rockwell S Glazer PM Decreased expression of the DNA mismatch repair gene Mlh1 under hypoxic stress in mammalian cells Mol Cell Biol 2003 4 3265 3273 10.1128/MCB.23.9.3265-3273.2003 12697826 PMC153206

[B77] Banath JP Sinnott L Larrivee B MacPhail SH Olive PL Growth of V79 cells as xenograft tumors promotes multicellular resistance but does not increase spontaneous or radiation-induced mutant frequency Radiat Res 2005 4 733 744 10.1667/3474.1 16296879

[B78] Koshiji M To KK Hammer S Kumamoto K Harris AL Modrich P Huang LE HIF-1alpha induces genetic instability by transcriptionally downregulating MutSalpha expression Mol Cell 2005 4 793 803 10.1016/j.molcel.2005.02.015 15780936

[B79] Fischer U Radermacher J Mayer J Mehraein Y Meese E Tumor hypoxia: impact on gene amplification in glioblastoma Int J Oncol 2008 4 509 515 18695880

[B80] Rodriguez-Jimenez FJ Moreno-Manzano V Lucas-Dominguez R Sanchez-Puelles JM Hypoxia causes downregulation of mismatch repair system and genomic instability in stem cells Stem Cells 2008 4 2052 2062 10.1634/stemcells.2007-1016 18511603

[B81] Lee JH Choi IJ Song DK Kim DK Genetic instability in the human lymphocyte exposed to hypoxia Cancer Genet Cytogenet 2010 4 83 88 10.1016/j.cancergencyto.2009.09.002 19963140

[B82] Kumareswaran R Ludkovski O Meng A Sykes J Pintilie M Bristow RG Chronic hypoxia compromises repair of DNA double-strand breaks to drive genetic instability J Cell Sci 2012 4 189 199 10.1242/jcs.092262 22266907

[B83] Mondello C Smirnova A Giulotto E Gene amplification, radiation sensitivity and DNA double-strand breaks Mutat Res 2010 4 29 37 10.1016/j.mrrev.2010.01.008 20093194

[B84] Popp HD Bohlander SK Genetic instability in inherited and sporadic leukemias Genes Chromosomes Cancer 2010 4 1071 1081 10.1002/gcc.20823 20842730

[B85] Patel KJ Yu VP Lee H Corcoran A Thistlethwaite FC Evans MJ Colledge WH Friedman LS Ponder BA Venkitaraman AR Involvement of Brca2 in DNA repair Mol Cell 1998 4 347 357 10.1016/S1097-2765(00)80035-0 9660919

[B86] Yu VP Koehler M Steinlein C Schmid M Hanakahi LA van Gool AJ West SC Venkitaraman AR Gross chromosomal rearrangements and genetic exchange between nonhomologous chromosomes following BRCA2 inactivation Genes Dev 2000 4 1400 1406 10837032 PMC316655

[B87] Venkitaraman AR Linking the cellular functions of BRCA genes to cancer pathogenesis and treatment Annu Rev Pathol 2009 4 461 487 10.1146/annurev.pathol.3.121806.151422 18954285

[B88] Walsh CS Ogawa S Scoles DR Miller CW Kawamata N Narod SA Koeffler HP Karlan BY Genome-wide loss of heterozygosity and uniparental disomy in BRCA1/2-associated ovarian carcinomas Clin Cancer Res 2008 4 7645 7651 10.1158/1078-0432.CCR-08-1291 19047089 PMC2677417

[B89] Min J Choi ES Hwang K Kim J Sampath S Venkitaraman AR Lee H The Breast Cancer Susceptibility Gene BRCA2 Is Required for the Maintenance of Telomere Homeostasis J Biol Chem 2012 4 5091 5101 10.1074/jbc.M111.278994 22187435 PMC3281639

[B90] Johnson AB Barton MC Hypoxia-induced and stress-specific changes in chromatin structure and function Mutat Res 2007 4 149 162 10.1016/j.mrfmmm.2006.10.007 17292925 PMC1924842

[B91] Arlt MF Durkin SG Ragland RL Glover TW Common fragile sites as targets for chromosome rearrangements DNA Repair (Amst) 2006 4 1126 1135 10.1016/j.dnarep.2006.05.010 16807141

[B92] Schwartz M Zlotorynski E Goldberg M Ozeri E Rahat A le Sage C Chen BP Chen DJ Agami R Kerem B Homologous recombination and nonhomologous end-joining repair pathways regulate fragile site stability Genes Dev 2005 4 2715 2726 10.1101/gad.340905 16291645 PMC1283964

[B93] Ozeri-Galai E Schwartz M Rahat A Kerem B Interplay between ATM and ATR in the regulation of common fragile site stability Oncogene 2008 4 2109 2117 10.1038/sj.onc.1210849 17934520

[B94] Tanaka H Yao MC Palindromic gene amplification–an evolutionarily conserved role for DNA inverted repeats in the genome Nat Rev Cancer 2009 4 216 224 10.1038/nrc2591 19212324

[B95] Wilson DM 3rd Thompson LH Molecular mechanisms of sister-chromatid exchange Mutat Res 2007 4 11 23 10.1016/j.mrfmmm.2006.11.017 17157333

[B96] Avidor-Reiss T Gopalakrishnan J Building a centriole Curr Opin Cell Biol 2013 4 72 77 10.1016/j.ceb.2012.10.016 23199753 PMC3578074

[B97] Chan JY A clinical overview of centrosome amplification in human cancers Int J Biol Sci 2011 4 1122 1144 22043171 10.7150/ijbs.7.1122 PMC3204404

[B98] Duijf PH Benezra R The cancer biology of whole-chromosome instability Oncogene 2013 4 4727 4736 10.1038/onc.2012.616 23318433

[B99] Moser SC Bensaddek D Ortmann B Maure JF Mudie S Blow JJ Lamond AI Swedlow JR Rocha S PHD1 Links Cell-Cycle Progression to Oxygen Sensing through Hydroxylation of the Centrosomal Protein Cep192 Dev Cell 2013 4 381 392 10.1016/j.devcel.2013.06.014 23932902 PMC3757158

[B100] Bristow RG Ozcelik H Jalali F Chan N Vesprini D Homologous recombination and prostate cancer: a model for novel DNA repair targets and therapies Radiother Oncol 2007 4 220 230 10.1016/j.radonc.2007.04.016 17531338

[B101] Meng AX Jalali F Cuddihy A Chan N Bindra RS Glazer PM Bristow RG Hypoxia down-regulates DNA double strand break repair gene expression in prostate cancer cells Radiother Oncol 2005 4 168 176 10.1016/j.radonc.2005.06.025 16026872

[B102] Koshiji M Kageyama Y Pete EA Horikawa I Barrett JC Huang LE HIF-1alpha induces cell cycle arrest by functionally counteracting Myc EMBO J 2004 4 1949 1956 10.1038/sj.emboj.7600196 15071503 PMC404317

[B103] To KK Sedelnikova OA Samons M Bonner WM Huang LE The phosphorylation status of PAS-B distinguishes HIF-1alpha from HIF-2alpha in NBS1 repression EMBO J 2006 4 4784 4794 10.1038/sj.emboj.7601369 17024177 PMC1618093

[B104] Hayashi M Yoo YY Christensen J Huang LE Requirement of evading apoptosis for HIF-1alpha-induced malignant progression in mouse cells Cell Cycle 2011 4 2364 2372 10.4161/cc.10.14.16313 21654209 PMC3322472

[B105] Bindra RS Schaffer PJ Meng A Woo J Maseide K Roth ME Lizardi P Hedley DW Bristow RG Glazer PM Down-regulation of Rad51 and decreased homologous recombination in hypoxic cancer cells Mol Cell Biol 2004 4 8504 8518 10.1128/MCB.24.19.8504-8518.2004 15367671 PMC516750

[B106] Bindra RS Schaffer PJ Meng A Woo J Maseide K Roth ME Lizardi P Hedley DW Bristow RG Glazer PM Alterations in DNA repair gene expression under hypoxia: elucidating the mechanisms of hypoxia-induced genetic instability Ann N Y Acad Sci 2005 4 184 195 10.1196/annals.1339.049 16382054

[B107] Bindra RS Glazer PM Co-repression of mismatch repair gene expression by hypoxia in cancer cells: role of the Myc/Max network Cancer Lett 2007 4 93 103 10.1016/j.canlet.2006.12.011 17275176

[B108] Chan N Pires IM Bencokova Z Coackley C Luoto KR Bhogal N Lakshman M Gottipati P Oliver FJ Helleday T Contextual synthetic lethality of cancer cell kill based on the tumor microenvironment Cancer Res 2010 4 8045 8054 10.1158/0008-5472.CAN-10-2352 20924112 PMC2978949

[B109] Wouters BG Koritzinsky M Hypoxia signalling through mTOR and the unfolded protein response in cancer Nat Rev Cancer 2008 4 851 864 10.1038/nrc2501 18846101

[B110] Lu Y Chu A Turker MS Glazer PM Hypoxia-Induced Epigenetic Regulation and Silencing of the BRCA1 Promoter Mol Cell Biol 2011 4 3339 3350 10.1128/MCB.01121-10 21670155 PMC3147797

[B111] Crosby ME Kulshreshtha R Ivan M Glazer PM MicroRNA regulation of DNA repair gene expression in hypoxic stress Cancer Res 2009 4 1221 1229 10.1158/0008-5472.CAN-08-2516 19141645 PMC2997438

[B112] Chang CJ Yang JY Xia W Chen CT Xie X Chao CH Woodward WA Hsu JM Hortobagyi GN Hung MC EZH2 promotes expansion of breast tumor initiating cells through activation of RAF1-beta-catenin signaling Cancer Cell 2011 4 86 100 10.1016/j.ccr.2010.10.035 21215703 PMC3041516

[B113] Cao P Deng Z Wan M Huang W Cramer SD Xu J Lei M Sui G MicroRNA-101 negatively regulates Ezh2 and its expression is modulated by androgen receptor and HIF-1alpha/HIF-1beta Mol Cancer 2010 4 108 10.1186/1476-4598-9-108 20478051 PMC2881117

[B114] Tsuchimoto T Sakata K Someya M Yamamoto H Hirayama R Matsumoto Y Furusawa Y Hareyama M Gene expression associated with DNA-dependent protein kinase activity under normoxia, hypoxia, and reoxygenation J Radiat Res 2011 4 464 471 10.1269/jrr.10137 21905307

[B115] Wirthner R Wrann S Balamurugan K Wenger RH Stiehl DP Impaired DNA double-strand break repair contributes to chemoresistance in HIF-1 alpha-deficient mouse embryonic fibroblasts Carcinogenesis 2008 4 2306 2316 10.1093/carcin/bgn231 18842680

[B116] Lara PC Lloret M Clavo B Apolinario RM Bordon E Rey A Falcon O Alonso AR Belka C Hypoxia downregulates Ku70/80 expression in cervical carcinoma tumors Radiother Oncol 2008 4 222 226 10.1016/j.radonc.2008.07.018 18706726

[B117] He F Li L Kim D Wen B Deng X Gutin PH Ling CC Li GC Adenovirus-mediated expression of a dominant negative Ku70 fragment radiosensitizes human tumor cells under aerobic and hypoxic conditions Cancer Res 2007 4 634 642 10.1158/0008-5472.CAN-06-1860 17234773

[B118] Sprong D Janssen HL Vens C Begg AC Resistance of hypoxic cells to ionizing radiation is influenced by homologous recombination status Int J Radiat Oncol Biol Phys 2006 4 562 572 10.1016/j.ijrobp.2005.09.031 16343804

[B119] Bindra RS Gibson SL Meng A Westermark U Jasin M Pierce AJ Bristow RG Classon MK Glazer PM Hypoxia-induced down-regulation of BRCA1 expression by E2Fs Cancer Res 2005 4 11597 11604 10.1158/0008-5472.CAN-05-2119 16357170

[B120] Madan E Gogna R Pati U p53Ser15 Phosphorylation disrupts p53-RPA70 complex and induces RPA70-mediated DNA repair in hypoxia Biochem J 2012 4 811 820 10.1042/BJ20111627 22288499

[B121] Hoffelder DR Luo L Burke NA Watkins SC Gollin SM Saunders WS Resolution of anaphase bridges in cancer cells Chromosoma 2004 4 389 397 15156327 10.1007/s00412-004-0284-6

[B122] Wimmer K Etzler J Constitutional mismatch repair-deficiency syndrome: have we so far seen only the tip of an iceberg? Hum Genet 2008 4 105 122 10.1007/s00439-008-0542-4 18709565

[B123] Li J Koike J Kugoh H Arita M Ohhira T Kikuchi Y Funahashi K Takamatsu K Boland CR Koi M Hemmi H Down-regulation of MutS homolog 3 by hypoxia in human colorectal cancer Biochim Biophys Acta 2012 4 889 899 10.1016/j.bbamcr.2012.01.017 22343000 PMC3793328

[B124] Grandori C Cowley SM James LP Eisenman RN The Myc/Max/Mad network and the transcriptional control of cell behavior Annu Rev Cell Dev Biol 2000 4 653 699 10.1146/annurev.cellbio.16.1.653 11031250

[B125] Nakamura H Tanimoto K Hiyama K Yunokawa M Kawamoto T Kato Y Yoshiga K Poellinger L Hiyama E Nishiyama M Human mismatch repair gene, MLH1, is transcriptionally repressed by the hypoxia-inducible transcription factors, DEC1 and DEC2 Oncogene 2008 4 4200 4209 10.1038/onc.2008.58 18345027

[B126] Nouspikel T DNA repair in mammalian cells : Nucleotide excision repair: variations on versatility Cell Mol Life Sci 2009 4 994 1009 10.1007/s00018-009-8737-y 19153657 PMC11131503

[B127] Rezvani HR Mahfouf W Ali N Chemin C Ged C Kim AL de Verneuil H Taieb A Bickers DR Mazurier F Hypoxia-inducible factor-1alpha regulates the expression of nucleotide excision repair proteins in keratinocytes Nucleic Acids Res 2010 4 797 809 10.1093/nar/gkp1072 19934262 PMC2817476

[B128] Filippi S Latini P Frontini M Palitti F Egly JM Proietti-De-Santis L CSB protein is (a direct target of HIF-1 and) a critical mediator of the hypoxic response EMBO J 2008 4 2545 2556 10.1038/emboj.2008.180 18784753 PMC2567410

[B129] Kitao H Takata M Fanconi anemia: a disorder defective in the DNA damage response Int J Hematol 2011 4 417 424 10.1007/s12185-011-0777-z 21331524

[B130] Kuhnert VM Kachnic LA Li L Purschke M Gheorghiu L Lee R Held KD Willers H FANCD2-deficient human fibroblasts are hypersensitive to ionising radiation at oxygen concentrations of 0% and 3% but not under normoxic conditions Int J Radiat Biol 2009 4 523 531 10.1080/09553000902883810 19466639 PMC3758881

[B131] Ramaekers CH van den Beucken T Meng A Kassam S Thoms J Bristow RG Wouters BG Hypoxia disrupts the Fanconi anemia pathway and sensitizes cells to chemotherapy through regulation of UBE2T Radiother Oncol 2011 4 190 197 10.1016/j.radonc.2011.05.059 21722982

[B132] Sun JD Liu Q Wang J Ahluwalia D Ferraro D Wang Y Duan JX Ammons WS Curd JG Matteucci MD Hart CP Selective tumor hypoxia targeting by hypoxia-activated prodrug TH-302 inhibits tumor growth in preclinical models of cancer Clin Cancer Res 2012 4 758 770 10.1158/1078-0432.CCR-11-1980 22184053

[B133] van den Beucken T Magagnin MG Jutten B Seigneuric R Lambin P Koritzinsky M Wouters BG Translational control is a major contributor to hypoxia induced gene expression Radiother Oncol 2011 4 379 384 10.1016/j.radonc.2011.05.058 21719133

[B134] Zhao H Luoto KR Meng AX Bristow RG The receptor tyrosine kinase inhibitor amuvatinib (MP470) sensitizes tumor cells to radio- and chemo-therapies in part by inhibiting homologous recombination Radiother Oncol 2011 4 59 65 10.1016/j.radonc.2011.08.013 21903282

[B135] Matsumoto S Batra S Saito K Yasui H Choudhuri R Gadisetti C Subramanian S Devasahayam N Munasinghe JP Mitchell JB Krishna MC Antiangiogenic agent sunitinib transiently increases tumor oxygenation and suppresses cycling hypoxia Cancer Res 2011 4 6350 6359 10.1158/0008-5472.CAN-11-2025 21878530 PMC3196374

[B136] Kaelin WG Jr The concept of synthetic lethality in the context of anticancer therapy Nat Rev Cancer 2005 4 689 698 10.1038/nrc1691 16110319

[B137] Chalmers AJ Lakshman M Chan N Bristow RG Poly(ADP-ribose) polymerase inhibition as a model for synthetic lethality in developing radiation oncology targets Semin Radiat Oncol 2010 4 274 281 10.1016/j.semradonc.2010.06.001 20832020

[B138] Hegan DC Lu Y Stachelek GC Crosby ME Bindra RS Glazer PM Inhibition of poly(ADP-ribose) polymerase down-regulates BRCA1 and RAD51 in a pathway mediated by E2F4 and p130 Proc Natl Acad Sci U S A 2010 4 2201 2206 10.1073/pnas.0904783107 20133863 PMC2836641

[B139] Rajesh C Baker DK Pierce AJ Pittman DL The splicing-factor related protein SFPQ/PSF interacts with RAD51D and is necessary for homology-directed repair and sister chromatid cohesion Nucleic Acids Res 2011 4 132 145 10.1093/nar/gkq738 20813759 PMC3017596

[B140] Feng Z Scott SP Bussen W Sharma GG Guo G Pandita TK Powell SN Rad52 inactivation is synthetically lethal with BRCA2 deficiency Proc Natl Acad Sci U S A 2011 4 686 691 10.1073/pnas.1010959107 21148102 PMC3021033

[B141] McEllin B Camacho CV Mukherjee B Hahm B Tomimatsu N Bachoo RM Burma S PTEN loss compromises homologous recombination repair in astrocytes: implications for glioblastoma therapy with temozolomide or poly(ADP-ribose) polymerase inhibitors Cancer Res 2010 4 5457 5464 10.1158/0008-5472.CAN-09-4295 20530668 PMC2896430

[B142] Fraser M Zhao H Luoto KR Lundin C Coackley C Chan N Joshua AM Bismar TA Evans A Helleday T Bristow RG PTEN deletion in prostate cancer cells does not associate with loss of RAD51 function: implications for radiotherapy and chemotherapy Clin Cancer Res 2012 4 1015 1027 10.1158/1078-0432.CCR-11-2189 22114138 PMC3378487

[B143] Martin SA McCabe N Mullarkey M Cummins R Burgess DJ Nakabeppu Y Oka S Kay E Lord CJ Ashworth A DNA polymerases as potential therapeutic targets for cancers deficient in the DNA mismatch repair proteins MSH2 or MLH1 Cancer Cell 2010 4 235 248 10.1016/j.ccr.2009.12.046 20227038 PMC2845806

[B144] Martin SA Hewish M Sims D Lord CJ Ashworth A Parallel high-throughput RNA interference screens identify PINK1 as a potential therapeutic target for the treatment of DNA mismatch repair-deficient cancers Cancer Res 2011 4 1836 1848 10.1158/0008-5472.CAN-10-2836 21242281

